# Ultrasound-Guided Biopsy of Pleural-Based Pulmonary Lesions by Injection of Contrast-Enhancing Drugs

**DOI:** 10.3389/fphar.2019.00960

**Published:** 2019-09-03

**Authors:** Ying Fu, Yuan-Yuan Zhang, Li-Gang Cui, Shi Tan, Yan Sun

**Affiliations:** Department of Ultrasound, Peking University Third Hospital, Beijing, China

**Keywords:** contrast media, lung neoplasms, biopsy, diagnostic imaging, sonography

## Abstract

In this study, a total of 58 patients with single subpleural pulmonary lesions (males: 36, females: 22, mean age: 63 ± 16.2 years) who underwent contrast-enhanced ultrasonography (CEUS) and had a definite diagnosis (benign lesions:25, malignant lesions:33) were enrolled. The number of biopsies, diagnostic accuracy rate, and the incidence of complications were recorded. The nodules were divided into two size subgroups: ≥5 cm (group 1), and <5 cm (group 2). The display rate of internal necrosis and change of pre-scheduled puncture paths were compared between subgroups. Also, the arrival times, intensity and uniformity of enhancement after the contrast agent injection, as well as the display rate of internal necrosis were recorded and compared between malignant and benign lesions. Finally, the average number of punctures was 2.9 ± 0.7 times. The total diagnosis rate was 98.3%. Local pneumothorax occurred in 2 patients. Hemoptysis occurred in 1 patient. No serious complications occurred. Internal necrosis was demonstrated in 20 of 58 lesions (34.5%). Sixteen of them had changed the planned puncture path due to the large necrosis area (80%, 16/20). For lesions in group 1, necrosis was found in 15 lesions and there was a statistically significant difference in the necrosis rate between the two subgroups (15/26 vs 5/32, *p* = 0.001). The change in the pre-scheduled puncture path occurred in 12 patients in group 1 while 4 patients in group 2 exhibited a change in the planned puncture path (*p* = 0.004). There was a statistically significant difference in the arrival times and intensity of enhancement between benign and malignant lesions (*p* < 0.05). In conclusion, CEUS guided biopsy is an effective, sensitive, and safe method for the diagnosis of pleural-based pulmonary lesions by facilitating a distinction between necrosis and active tissue. The current findings indicated that CEUS before a biopsy may be especially vital in lesions ≥5 cm.

## Introduction

Lung cancer is a primary cancer that has become a major public health concern. It is the most frequent cause of cancer-related mortality in men, and lung cancer-related morbidity and mortality are both increasing (Ferlay et al., 2015). The detection rate of peripheral pulmonary lesions has increased in the recent years with the application of computed tomography (CT), especially multi-slice spiral CT, in the diagnosis of lung cancer (Swensen, 2002). Clinically, peripheral pulmonary lesions are primarily diagnosed on the basis of exfoliative sputum cytology, fiberoptic bronchoscopy, bronchoalveolar lavage, medical thoracoscopy, and needle biopsy results (Ofiara et al., 2012). Ultrasound-guided percutaneous lung biopsy has a success rate similar to that obtained with CT guidance, a lower complication rate, and a shorter operation time; also, it is more economical, does not involve radiation, and offers a more convenient puncture guidance method. This technique has gained wide acceptance in clinical conditions and shows satisfactory puncture results (Khosla et al., 2016). However, conventional ultrasonography cannot distinguish between atelectatic lung tissue and necrotic lesions, which often results in false-negative biopsy results that can confound clinical diagnosis and treatment and cause complications (Wang et al., 2015).

The new ultrasound contrast agents that have emerged in recent years have greatly increased visualization of the microcirculation on ultrasonography. SonoVue (Bracco SpA, Milan, Italy) is a second-generation ultrasound contrast agent that contains sulfur hexafluoride as a low-solubility inert gas phase surrounded by a layer of phospholipids. The microbubbles have an average diameter of 2.5 µm, and 90% of the microbubbles have a diameter of less than 6 µm. After intravenous injection, the contrast agent microbubbles enter the arterial system through the pulmonary circulation barrier and participate in systemic circulation, greatly enhancing the backscattering of sound waves. This, combined with low mechanical index ultrasound contrast imaging technology, permits a sensitive, real-time determination of tissue microcirculation and can distinguish between living areas and necrotic foci in tissues. Currently, this technique is widely used for guidance in needle biopsies of liver and thyroid nodules and has yielded satisfactory results (Wu et al., 2006; Li and Luo, 2013; Eso et al., 2016).

Lungs have a dual blood supply, which provides a pathophysiological basis for using ultrasonography to distinguish between benign and malignant lesions. The pulmonary artery blood supply phase is observed 2–6 s after the injection of a contrast agent and the bronchial artery blood supply phase is observed 7–20 s after injection (Görg et al., 2006a). Most malignant lesions are supplied by the bronchial artery, so the time phase can be used to distinguish lesions originating from lung tissue or the bronchial blood supply, thereby avoiding an unnecessary needle biopsy (Görg et al., 2006b). Contrast-enhanced ultrasonography (CEUS) can also effectively avoid necrotic tissue inside the lesion and atelectatic lung tissue at the periphery of the lesion, thereby accurately guiding the biopsy.

Previously published reports of CEUS guidance for peripheral pulmonary lesions have focused on the differences between contrast-enhanced guidance and conventional ultrasound guidance. However, they did not focus on the effects of lesion size. Thus, the purpose of our study is to examine the diagnostic efficacy of CEUS guidance for needle biopsies of peripheral pulmonary lesions, with an emphasis on the effects of nodule size on contrast-enhanced guidance.

## Materials and Methods

### Patients

A total of 58 patients with peripheral pulmonary lesions diagnosed by chest CT and confirmed by pathological assessments between May 2016 and May 2018 were selected for the study. The study population consisted of 36 males and 22 females, the average age was 63 ± 16.2 years, and the average lesion size was 4.4 ± 2.7 cm. Written, informed consent was obtained from all patients prior to the CEUS and ultrasound-guided percutaneous lung biopsy. This retrospective study has been reviewed by the Institutional Review Board of Peking University Third Hospital.

### Ultrasound Contrast Agent and Ultrasound Examination Procedure

All included patients were examined by CT to confirm the location of the lesions. Next, two-dimensional ultrasonography was performed to record its location and size, and conventional color Doppler ultrasonography was used to determine blood flow distribution. An ultrasound specialist with 10 years of experience marked possible intercostal puncture sites and planned needle paths. Next, a CEUS examination was performed, and the size of the lesion, the target puncture region, and the presence or absence of perfusion defects as well as their range were confirmed on the basis of lesion perfusion. This information was recorded and discussed by two physicians experienced in puncture biopsy based on the CEUS results. Once a consensus was reached, a new needle path was planned.

The ultrasound contrast agent SonoVue (Bracco, Italy), which contains sulfur hexafluoride (SF_6_) enclosed in phospholipid microcapsules, was used. It was dissolved and mixed thoroughly with 5 mL normal saline, and 2.4 mL (5 mg/ml concentration, 12 mg SF_6_/patient) was rapidly injected into the body through the superficial cephalic vein over 2–3 s each time. The GE Logiq 9 (GE Healthcare, Milwaukee, WI) ultrasound instrument was used with a C5-1 probe at a probe frequency of 3.0–5.0 MHz. The instrument was initialized with a low mechanical index (MI: 0.11–0.13). A timer was started at the same time the contrast agent was injected, and the changes in enhanced perfusion of the lesion were observed in real time. After imaging, the size and position of the lesions were carefully recorded based on video data. Contrast-enhanced and non-contrast-enhanced regions and their adjacencies were confirmed by observing contrast agent perfusions and the process of enhancement in the lesion. Solid enhancement regions were identified as targets for punch biopsies, and needle insertion points, needle paths, biopsy target regions, and sample lengths were confirmed. A needle biopsy was performed within 30–60 min after imaging.

Diagnosis using CEUS was performed by a physician with 5 years of experience in this technique. The main descriptive indicators included were the time of the contrast agent’s arrival to the lesion on imaging, the time of its arrival to peripheral lung tissue or the intercostal artery, the uniformity of lesion enhancement, and the peak intensity. The time of arrival to the lesion on imaging refers to the time at which the lesion is filled with the contrast agent. The time of arrival to the lung tissue is the first time when the contrast agent microbubbles fill the peripheral lung tissue. The time of arrival to the intercostal artery is the time at which the contrast agent fills the intercostal artery. The difference in arrival times between the lesion and the lung tissue was calculated. The enhancement time was close to the bronchial artery if it was greater than 3 s and close to the pulmonary artery if it was 3 s or less. The uniformity within the lesion was defined as the uniformity and consistency of the filling of the lesion with contrast agent; low uniformity indicated that the lesion was not uniformly enhanced. Peak intensity was divided into level-high enhancement and low enhancement. The reference was the degree of enhancement of the peripheral lung tissue. Enhancement equal to or higher than that of the lung tissue was deemed level-high enhancement, and enhancement lower than that of the lung tissue was deemed low enhancement.

### Needle Biopsy Indications and Biopsy Method

Inclusion criteria for CEUS guidance were as follows: imaging confirms that the lesion is located at the periphery of the lungs and is visible on ultrasonography, a signed informed consent form is acquired, and no contraindications are suggested based on the instructions of the ultrasound contrast agent. Exclusion criteria were as follows: coagulopathy or bleeding diathesis, biopsy intolerance due to cardiopulmonary insufficiencies or a severe cough, the lesion is not clearly visible on ultrasonography, allergy to ultrasound contrast agents, and refusal of CEUS.

After confirming the needle path, a conventional aseptic drape was applied. Local anesthesia was achieved by injection of 1% lidocaine (Liduokayin; Yimin Pharmaceutical Co., Ltd, Beijing, China) from the upper edge of the rib between the predetermined intercostals. The patient was asked to hold his or her breath, and the biopsy needle was used to rapidly puncture the tumor margin under ultrasound guidance. The Bard automatic biopsy gun (Bard, USA) was equipped with an 18-gauge biopsy needle. The biopsy gun had a range of 15–22 mm. Areas in the lesion with significant enhancement based on imaging conditions were selected for biopsy. Collected tissues were fixed in a 10% formalin solution. Biopsies were collected 1–4 times as tolerated by the patient.

After the operation, the patient’s vital signs and hemoptysis were closely observed. Follow-up complications such as hemothorax, pneumothorax, and chest pain were observed. Patient with uncomfortable symptoms underwent same-day X-ray examinations to promote the early detection of complications such as pneumothorax.

### Pathological Diagnosis and Follow-Up

Two pathologists with 10 years of experience read images together and made a final determination. Benign lesions were followed up for more than 6 months to confirm shrinkage or that they did not enlarge before being diagnosed as benign. If tissue collection was insufficient, a pathological diagnosis could not be made, and the puncture biopsy was considered to have failed and needed to be re-performed. Some patients who had undergone surgical resection, such as patients whose diagnoses were not consistent with the diagnosis from surgical resection specimens, were subject to postoperative pathological diagnosis.

### Observational Indices

Several indices were used in our analysis. First, the lesion time of arrival on imaging was recorded as close to the pulmonary artery or close to the bronchial artery. In addition, the uniformity of lesion enhancement, the peak intensity, presence of necrosis inside the lesion, and changes in the originally planned needle path a result of CEUS were recorded. Second, the success rate of ultrasound-guided percutaneous lung puncture biopsy and the incidence of post-biopsy complications were recorded. Third, based on their size, lesions were classified as either less than 5 cm or greater than or equal to 5 cm, and the necrosis rate and needle path change rate in the two groups were compared.

### Statistical Analysis

SPSS 21.0 statistical software (IBM Corporation, Armonk, NY) was used. Continuous data are shown as the mean ± standard deviation (x ± s). The χ2 test was used for pairwise comparisons of discrete data. The independent or paired samples *t*-test was used for comparisons of differences in lesion size. Differences with *p* < 0.05 were considered statistically significant.

## Results

### Number of Biopsy Puncture Attempts and Diagnostic Accuracy Rate

The average frequency of puncture attempts was 2.9 ± 0.7 times, and the average tissue length was 1.2 ± 0.4 cm. Pathological assessments indicated benign lesions in 25 cases and malignant lesions in 33 cases. The specific pathological types are shown in **Table 1**.

**Table 1:** Pathological types of the cases.

Pathological type	Number of cases
Benign	25
Inflammation	12
Tuberculosis	5
Mycosis	2
Organizing pneumonia	6
Malignant	33
Adenocarcinoma	23
Squamous cell carcinoma	4
Small cell lung carcinoma	1
Lymphoma	1
Metastatic cancer	4

**Figure 1:**
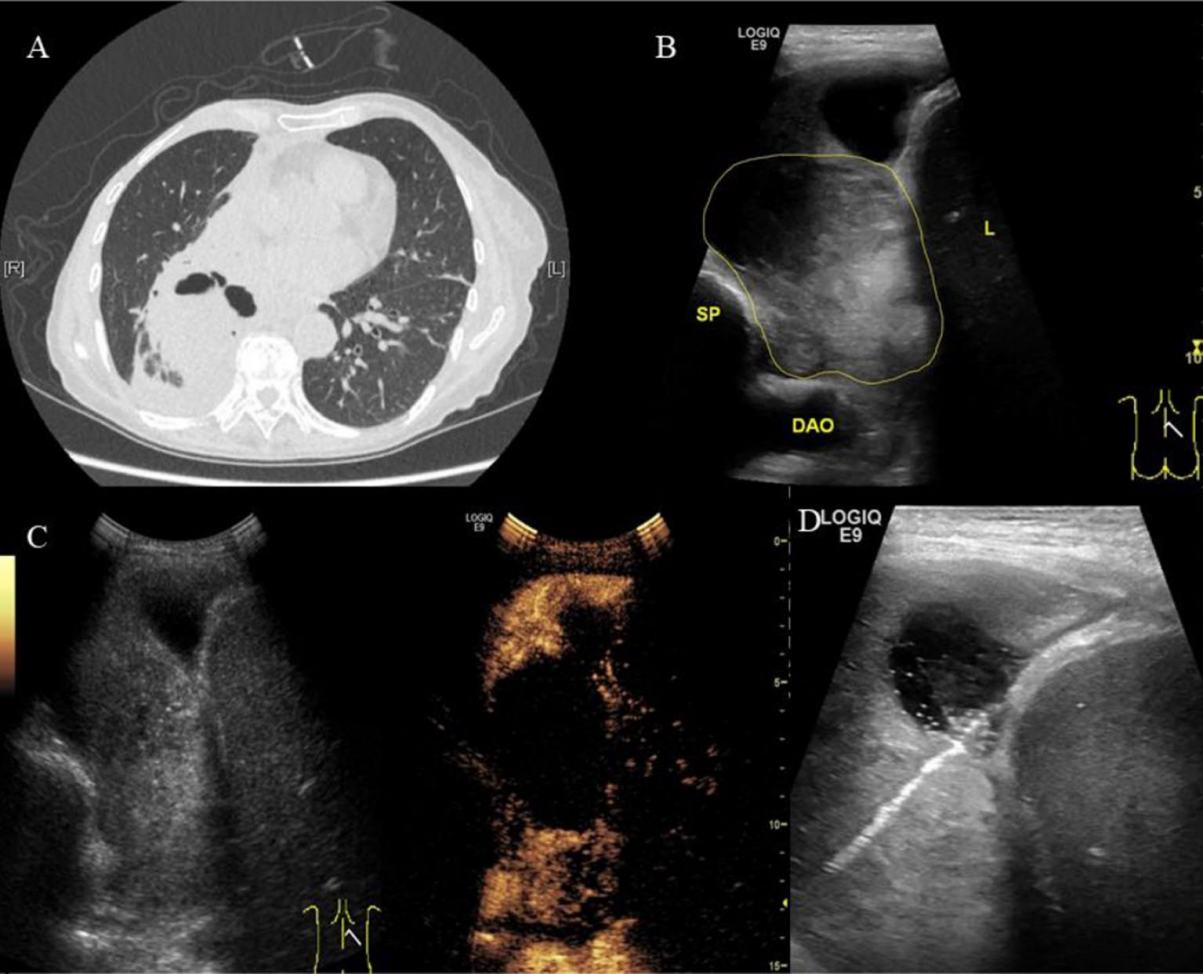
A 70-year-old female patient was admitted to our hospital with cough, hemoptysis and fever. The representative CT image showed lung lesion in right lower lobe. **(A)** An elliptical mass with soft tissue density was presented in the basal segment of the right lower lobe, the boundary was unclear. Low density and the gas–liquid plane was observed in central region of the mass. Atelectasis was observed around the mass. **(B)** Two-dimensional ultrasound showed a hypoechoic solid mass with diameter of about 6 cm (the yellow line). The boundary of the mass was blurred. A small amount of pleural fluid was observed around the mass. DAO, descending aorta; SP, spine; L, liver. **(C)** Contrast-enhanced ultrasound showed a significantly inhomogeneous enhancement of mass. Uniform enhancement was observed in the superficial part of the mass at 9 s without enhancement in the deep part of the mass. However, the deep part of the mass was inhomogeneously enhanced at 13 s, showing a large non-enhanced necrotic area. **(D)** The superficial part of the mass may represent the atelectatic lung tissue. The necrotic tissue and alive tumor tissue respectively located in the middle and deep parts. Therefore, we targeted the deep part of the mass as puncture area for biopsy. The arrow indicated the puncture needle. The biopsy specimen was white. The pathological findings were adenocarcinoma of the lung.

**Figure 2:**
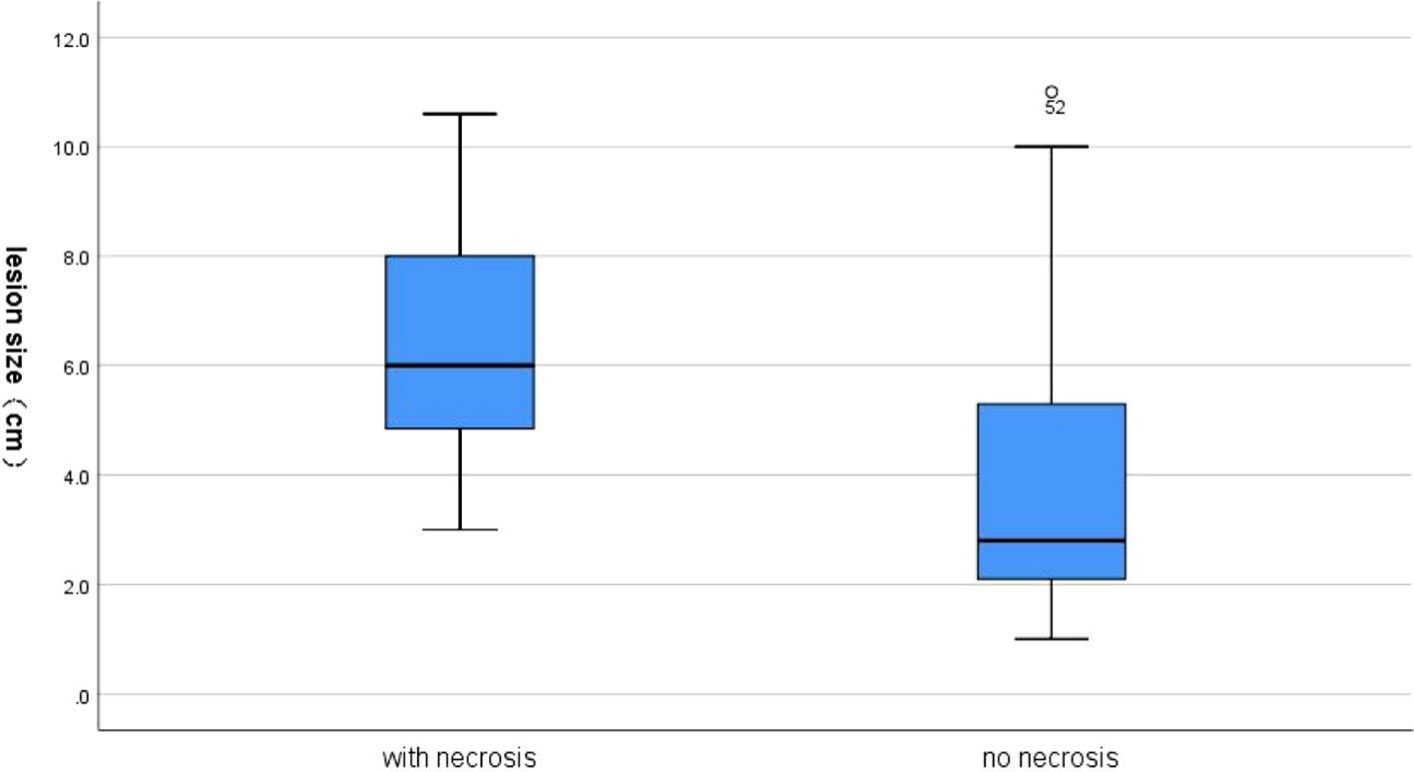
A box-plot between necrosis and lesion size.

Internal necrotic foci were found in 20 cases on imaging (20/58, 34.5%). Of these, the originally planned needle path was changed in 16 cases (27.6%) due to the large extent of necrosis (**Figure 1**). Necrosis was found in 15 of 26 cases of nodules that were 5 cm or larger (*p* = 0.001), of which the original needle path was changed in 12 cases (*p* = 0.004). Necrosis was found in 5 of the 32 cases of nodules less than 5 cm in size, of which the original needle path was changed in cases (**Tables 2** and **3**). **Figure 2** showed necrosis in 58 patients with different lesion size. **Table 4** showed the patient demographics and lesion characteristics.

**Table 2:** Necrosis in lesions of different sizes.

Group	Necrosis found	No necrosis found	*p*-value
>5 cm	15	11	0.001
<5 cm	5	27	
Total	20	38	

**Table 3:** Changes in the original puncture path in lesions of different sizes.

Group	Puncture path changed	No puncture path change	*p*-value
>5 cm	12	14	0.004
<5 cm	4	28	
Total	16	42	

**Table 4:** Patient demographics and lesion characteristics.

Characteristics	Malignant	Benign	*p*-value
Sex			0.175
Male	18	18	
Female	15	7	
Median age(years)	60.0 ± 20.0	66 ± 12.0	0.46
Median size (cm)	5.0 ± 2.9	3.5 ± 2.4	0.59
Necrosis present	12	8	0.729
the originally planned needle path changed	9	7	0.951

The success rate of biopsy procedures guided by CEUS was 98.3% (57/58 cases). In only one case, the necrotic area was too large, and the pathological results of the puncture biopsy did not yield a clear conclusion. That patient underwent surgical resection and was found to have lung adenocarcinoma.

### Ultrasound Imaging Results

The average lesion size from imaging was 4.2 ± 2.8 cm, which was not significantly different from the size measured using 2-dimensional ultrasonography (*p* = 0.886). **Table 5** showed the imaging presentation of the lesions. The difference in enhancement time between benign and malignant cases was statistically significant (*p* = 0.003). The enhancement time was near the pulmonary artery in 88% of the benign cases, and only 3 of the 25 benign cases showed enhancement time near the bronchial artery. There was no statistically significant difference in the degree of uniformity and peak intensity between the two groups.

**Table 5:** Lesion enhancement of cases.

Enhancement	Number of cases			*p*-value
Time to enhancement	Total	Benign	Malignant	0.003
Near pulmonary artery	39	22	17	
Near bronchial artery	19	3	16	
Uniformity				0.512
Uniform	25	12	13	
Not uniform	33	13	20	
Peak intensity				
Level-high	42	18	24	0.004
Low	16	7	9	

### Complications

Local pneumothorax occurred in two cases and improved after conservative treatment. One patient developed hemoptysis after one needle puncture. The puncture was discontinued, and the pathology after puncture was confirmed. The complication rate was 5.17% (3/58), and no inflammatory complications occurred.

## Discussion

The aim of this study was to evaluate ultrasound-guided biopsies of pleural-based pulmonary lesions by injecting contrast-enhanced drugs. There were significant differences in the necrosis rate between different lesion sizes visible *via* CEUS. Therefore, a change in the pre-scheduled puncture path occurred more often in patients with lesions ≥5 cm, with statistical significance. The overall diagnosis rate was 98.3% with no serious complications. We can conclude that CEUS can distinguish necrosis and active tissue sensitively, which is helpful and crucial in improving the biopsy success rate. Furthermore, CEUS before a biopsy may be more important in lesions ≥5 cm.

Diagnostic techniques for lung cancer include chest radiography, exfoliative sputum cytology, fiberoptic bronchoscopy, and CT examination. However, the positive rates with these techniques are insufficient, and serum cancer marker measurements have not shown acceptable outcomes with respect to early diagnosis. Final diagnosis of many intrathoracic masses can only be made by a chest biopsy to collect lung tissue for pathological examination, but this is often rejected by patients and their families (Ofiara et al., 2012).

Ultrasound-guided peripheral lung puncture has recently gained gradual acceptance in clinical conditions. In comparison with traditional CT guidance, ultrasound guidance shows similar accuracy, but it does not involve radiation, is safe, can monitor the position of the needle tip at all times, can be performed at the bedside, and has a low cost. It is now widely employed in clinical practice and has been extended to the diagnosis of pleural lesions. The diagnostic rate achieved by this minimally invasive procedure is 66.6–78% (Sagar et al., 2000; Cao et al., 2011), with more false-negative findings that may be closely related to tumor size (Sartori et al., 2004). In a study of CT-guided biopsies, Yeow et al. (Yeow et al., 2003) analyzed 631 biopsy results and found that lesion sizes smaller than 1.5 cm and larger than 5 cm were closely associated with low diagnostic accuracy. The low diagnostic accuracy for lesions smaller than 1.5 cm can be easily explained by unsatisfactory sample collection through biopsy needles due to the small lesion size. In contrast, the increased necrotic regions in lesions larger than 5 cm are the main cause of false-negative findings in larger lesions. Although ultrasonography can distinguish obviously necrotic regions, not all necrotic regions are characterized as anechoic and are easily distinguished, and some necrotic regions are also hypoechoic or isoechoic (Sartori et al., 2004). Thus, 9–26% of the cases of nodules with large necrotic regions will yield insufficient data (Trevisani et al., 1994; Sagar et al., 2000). In addition, conventional ultrasonography has a limited ability to distinguish between tumor tissue and atelectatic lung tissue.

The recent emergence of a new generation of ultrasound contrast agents has greatly improved the ability of ultrasound technology to visualize the microcirculation in lesions. The new type of ultrasound contrast agent represented by SonoVue is a pure blood pool contrast agent. It has an average diameter of 2.5 µm, which is smaller than the diameter of red blood cells, so it remains in the circulation for a long time, does not enter the interstitial space, and has good stability in blood vessels. The microscopic distribution of microbubbles in small blood vessels in tissues can be more clearly observed, which is more conducive to studies of microvascular perfusion. Finally, these microbubbles are excreted from the body by respiration, so they offer the advantages of being harmless to the human body and being extremely safe, while providing good image development efficacy. Because the components contained in the microbubbles are non-toxic, they have no effect on the liver and kidneys. Ultrasound contrast can improve the effects of imaging, primarily in low mechanical index ultrasonic scanning. Vibration does not rupture the contrast agent microbubbles, and such situations can produce nonlinear effects. This technology can receive signals from the contrast agent microbubbles, filtering out harmonic signal interference from the tissue. The technique allows for the separation of signals from the contrast agent microbubbles and tissue, overcoming the limitation of traditional color Doppler ultrasonography, which shows insensitivity to low-velocity small blood vessels. Thus, this method can provide better information for a qualitative diagnosis of tumors on the basis of the blood supply characteristics of different tumors (Lim et al., 2004).

Currently, ultrasound contrast agents have been used in the differential diagnosis of lung lesions (Sperandeo et al., 2006; Dong et al., 2015; Bai et al., 2016). The natural blood supply to the lungs is a dual blood supply. The difference in tissue vascular anatomical structure and hemodynamics between benign and malignant lesions provides a pathophysiological basis for CEUS (Görg et al., 2006b) and can help distinguish atelectatic lung tissue and lung lesions through the time phase. Most researchers believe that the pulmonary artery blood supply is mainly responsible for gas exchange in the lungs, whereas neovascularization of malignant tumors is supplied by the bronchial artery (Görg et al., 2006a; Dong et al., 2015). Görg et al. found that the time to arrival of contrast agents in malignant lesions was significantly longer than that in benign lesions (Görg et al., 2006a). Caremani et al. proposed an arrival time of 10 s as the boundary for distinguishing between benign and malignant lesions but did not provide a statistical basis for this finding (Caremani et al., 2008). Wen et al. used 8 s as the boundary between benign and malignant lesions and considered a time of arrival of more than 8 s as an indication of bronchial artery blood supply, thus defined as a malignant lesion, but the diagnostic specificity was only 73.3% (Wen et al., 2013).

Due to the complexity underlying the physiological conditions in the human body, such as the physiological anastomosis between the pulmonary arterial and the bronchial arterial circulation, the intrapleural pressure gradient, cardiac function, examination position, and other factors will affect circulatory status. In addition, some artificial factors, such as the injection rate of contrast agents by different operators, also affect the time of arrival of the contrast agent to the lesion (Sperandeo et al., 2017). Therefore, relying solely on the time of arrival to the lesion cannot completely distinguish between benign and malignant lesions.

In the present study, the method of finding the difference in the times of arrival to the lesion and to the peripheral pulmonary tissue proposed by Bai et al. (2016) was used. This eliminates the differences in the time of arrival due to differences in the circulation of different individuals, thus effectively distinguishing between benign and malignant lesions. Because the study by Bai et al. used software to distinguish the time of arrival, the boundary value was 2.5 s. This observation was made visually in the present study, and thus the boundary value was set to 3 s, which also yielded informative results. It is worth noting that there were 17 cases of malignant lesions in this study with blood supply from the pulmonary artery (**Table 4**), which may be related to factors such as lesions being mixed with atelectasis. Therefore, relying solely on CEUS findings to distinguish between benign and malignant lesions is likely to produce false-negative findings, which has also been criticized in a recently published large-scale clinical study (Sperandeo et al., 2017). This is not the primary research purpose of this paper; instead, the study aimed to determine whether puncture biopsy can be effectively guided by enhancement after increased micro-perfusion.

In addition, although there were only two cases with a pulmonary lesion and atelectatic lung tissue in our group, the time to enhancement of atelectasis was shown to be significantly earlier than that of lung cancer (**Figure 1**). Also, the extent of enhancement was higher than lung cancer. This is consistent with the literature reports. Lei et al explored the clinical value of CEUS for biopsy in patients with central lung cancer with obstructive atelectasis (Lei et al., 2018). Enhancement of the central lung cancer mass began at 10–15 s, while that of 100 cases with atelectatic lung tissue began at 6–10 s and 12 cases at 10–15 s. The peak intensity was also higher in atelectasis for the contrast media may trapped in the lung tissue after having washed out of the blood pool. By analyzing the time to enhancement and extent of enhancement, CEUS can be used for differentiating atelectasis and lung cancer. CEUS can also distinguish atelectasis of different causes, mainly obstructive atelectasis and compression atelectasis. Görg et al studied 30 patients with atelectasis using CEUS. They found CEUS showed different patterns of enhancement in compression atelectasis and obstructive atelectasis. Compression atelectasis is characterized by a high specific CEUS pattern with a short time to enhancement and a marked extent of enhancement, indicating predominant pulmonary vascularization. In patients with obstructive atelectasis, a variable CEUS pattern was found (Görg et al, 2006b). Despite the small number of cases, CEUS shows a certain ability to identify and it deserves further study.

CEUS-guided tumor biopsy has been widely used in liver, mediastinal, and thyroid tumors. Fu et al. (2016) studied the value of CEUS in puncture biopsy of anterior mediastinal tumors. They concluded that CEUS guided technique could reliably differentiate the viable regions from necrotic tissues in a lesion and identify large superficial blood vessels in anterior medial mediastinal lesions before biopsy. Therefore, the safety and accuracy of puncture biopsy are improved.

Cao et al. (2011) conducted a comparative study of puncture biopsies of peripheral pulmonary lesions and mediastinal lesions. A diagnostic accuracy of 93.6% was achieved in 62 cases with ultrasound-guided biopsy, whereas a diagnostic accuracy of only 78% was achieved in 59 cases with conventional ultrasound guidance. The lesion necrosis rate in the CEUS group was 41.9%. The importance of CEUS in puncture biopsies of peripheral lung lesions is to distinguish between necrotic and active tissue; this was also verified in our study (necrosis rate: 34.5%). It is well known that necrotic tissue often develops in malignant lesions, and as tumor volume increases, the likelihood that necrotic tissue appears in the tumor increases. In the present study, a subgroup analysis was performed, which found that the necrosis rate in lesions 5 cm and larger was significantly higher than that in lesions smaller than 5 cm. In addition, the planned needle path was changed in most cases, indicating the necessity of preoperative CEUS for larger nodules.

In lung lesions, necrosis in benign tubercle lesions is common in addition to necrosis in malignant lesions. In the present study, four of the five patients with tuberculosis had necrosis confirmed by CEUS. Therefore, it is especially important to perform CEUS on peripheral pulmonary lesions before a puncture biopsy because it can clearly show necrotic regions without blood supply, and can also clearly depict active lesions with low-velocity blood supply. After CEUS, the position and adjacencies of the non-enhanced and enhanced regions within the lesion can be judged. During a puncture biopsy, the necrotic tissue can be avoided, and the enhanced regions with blood supply can be selected for a puncture biopsy, significantly improving the positive rate.

The advantage of CT-guided puncture biopsy is that it can puncture masses in various parts of the lung and is less restricted by location. Its disadvantages include the higher cost and the effects of radiation. The literature reports that the incidence of pneumothorax with CT guidance can reach 27–54% (Klein et al., 1996; Collings et al., 1999). The advantages of ultrasonography and CEUS guidance are that the whole puncture biopsy process is performed under direct vision. It allows for the observation of lung movements in real time, and instantly allows the patient to hold the needle after holding his or her breath, thereby facilitating the avoidance of structures such as gas-filled lungs, blood vessels, nerves, or necrotic tissue. It has low operational difficulty, can be completed in a short time, is safe and accurate, and has a low incidence of complications. Although the incidence of complications in the present study was 5.17%, they were all minor complications, and the patients improved after conservative treatment. In other studies, the incidence of complications of CEUS guidance in peripheral pulmonary disease was 3.3% or less (Cao et al., 2011; Dong et al., 2015; Wang et al., 2015). The reduced occurrence of complications may be related to the fact that the ultrasound techniques described above can monitor local conditions in real time. In addition, the low complication rate is related to the currently small number of cases of CEUS-guided pulmonary nodule puncture biopsies.

The present study has the following shortcomings: the number of cases is relatively small and the nature of the study was retrospective. However, the results of this study may greatly inspire interest, and participation, among clinicians regarding ultrasound-guided peripheral pulmonary nodule puncture techniques and provide the foundation for future large-scale prospective clinical studies.

## Conclusion

CEUS-guided peripheral pulmonary lesion biopsy is a safe and effective technique with high patient and clinical acceptance. It can effectively distinguish between necrotic and active tissue, atelectatic lung tissue, tumor tissue, etc., thus improving the success rate of puncture biopsy. CEUS is particularly important for needle biopsies of lung lesions that are 5 cm or larger.

## Ethics Statement

This study was carried out in accordance with the recommendations of the Institutional Review Board of Peking University Third Hospital, with written informed consent form all subjects. All subjects gave written informed consent in accordance with the Declaration of Helsinki. The protocol was approved by the Institutional Review Board of Peking University Third Hospital.

## Author Contributions

YF drafted the manuscript, coordinated data collection, analyzed the data, and reported the study results. L-GC developed the study protocol, revised the manuscript, supported in data collection, analyzed the data, and reported the study results. Y-YZ, ST, YS participated in the design of the study and revised the manuscript. All authors read and approved the final manuscript.

## Conflict of Interest Statement

The authors declare that the research was conducted in the absence of any commercial or financial relationships that could be construed as a potential conflict of interest.
